# A Parenting Behavior Intervention (the Strengthening Families Program) for Families: Noninferiority Trial of Different Program Delivery Methods

**DOI:** 10.2196/14751

**Published:** 2019-11-18

**Authors:** Karol Linda Kumpfer, Jaynie Litster Brown

**Affiliations:** 1 Health Sciences Center University of Utah Salt Lake City, UT United States; 2 Strengthening Families Program LLC Salt Lake City, UT United States

**Keywords:** parenting, youth drug prevention, family skills, DVD, Strengthening Families Program, internet, noninferiority trial

## Abstract

**Background:**

The Strengthening Families Program (SFP) is an evidence-based parent training and youth life skills and drug prevention program traditionally delivered in group settings. Families attend parent and youth classes conducted by trained facilitators. Recently, a 2-disk home-use DVD series was created with the same SFP skills as the group classes for parents and the youth to watch together at home. Additional lesson material was added that included healthy brain development, school success, anger management, dangers of alcohol and drugs, and mindfulness. The SFP DVD reduces SFP delivery costs for agencies and logistic burdens to families. Creative applications of the DVD include holding SFP DVD family discussion groups of multiple families and using SFP DVD video clips as part of a shorter 10-week group class version for parents and the youth.

**Objective:**

This study aimed to examine three different DVD implementation scenarios using a noninferiority trial, contrasting target outcomes with an age-matched sample culled from a national norm database of families who completed a standard SF*P* 14-week class.

**Methods:**

The partial eta-square was used to compare effect sizes between the different delivery modalities for relevant programmatic outcomes. We adjusted the effect sizes by demographic measures to determine whether there were site-specific features influencing program outcomes.

**Results:**

For the unadjusted effect size comparisons, 13 of the 15 indicated that the home-use DVD outperformed group norms with an average 0.13 effect size estimate difference across the comparisons (28% improvement in the effect size for DVD condition). Comparisons of the home-use DVD condition with the mixed DVD use conditions showed no discernable pattern where one condition consistently outperformed another. Adjusted effect sizes still reinforced the superiority of the DVD conditions; however, there was some shrinkage in the effect sizes as expected with the inclusion of relevant covariates.

**Conclusions:**

The home-use DVD shows that it is possible to effectively deliver an affordable family-based intervention using alternative technology outside of the traditional group-based class format. In almost all of the comparisons, the DVD conditions outperformed the group norms, underscoring that low-cost DVDs or viewing the videos on the Web may provide a useful surrogate for costly group-based formats. Future studies may want to improve on the quasi-experimental design by examining programmatic differences based on delivery format using a randomized controlled trial, thus strengthening the causal framework regarding program effects. In addition, the assessment protocol relied on retrospective reporting, which, although this can limit response shift bias, does not separate data collection in time as with a true pre- and posttest design.

## Introduction

### Background

Adolescence is a critical period of brain development, with a vulnerability for neurotoxic substances, such as alcohol and drugs [1,2], and a high risk for addiction [3]. However, in the 2018 Monitoring the Future survey, 30% of 12th-grade students reported drinking [4]. Parenting skills and youth drug prevention programs have, therefore, become a widely used antidote to youth alcohol and drug use. Several reviews have shown this modality of prevention to be effective following rigorous efficacy trials conducted by independent research teams in different settings and with different populations [5-7]. The Strengthening Families Program (SFP) offers parenting skills training in combination with youth life skills and drug prevention. It is an evidence-based family skills training program with consistent evidence of effectiveness obtained from randomized controlled trials (RCTs) and quasi-experimental replications spanning >30 years [8]. The program’s theoretical base is the tested Social Ecology Model [9] detailing mechanisms through which risk and protective factors contribute to adolescent drug use and delinquency [10]. SFP harnesses the vital role played by parents in the socialization of their children [11,12]. Hallmark features of SFP family skills training include reinforcing the need for loving family bonds, setting clear rules against antisocial behavior, and ensuring that parents supervise their child’s activities. These skills help prevent rule transgressions and avoid instigation of delinquency through the formation of deviant peer bonds.

SFP was originally tested and found effective in a 4-condition dismantling RCT with substance-abusing caretakers of children aged 6 to 11 years [13,14]. This original RCT found that combining parents, family, and children’s skills training produced the best results. In its traditional group-delivered instructional format, the highly structured program usually begins with dinner and involves 14 weekly sessions, with separate 1-hour skills training sessions for the parent and youth followed by a joint family skills practice session. Delivery is conducted by gender-balanced and culturally sensitive family coaches who are trained to teach and reinforce newly acquired skills. Cost to deliver the 14-week program is about US $1000 per family, depending on personnel fees and the amount spent on attendance incentives (eg, food and transportation).

The program has been tested with parents with children of varying ages [15,16] in urban [17] and rural populations [18,19] and demonstrated to be culturally sensitive for most groups and local customs [20,21]. International applications have included effectiveness trials conducted in Ireland [22] and Thailand [23]. A shorter, 7-session version, the Iowa SFP (ISF*P* 10-14) was created for low-risk families as part of a collaborative partnership with Dr. Kumpfer and investigators at Iowa State University and has been tested in several randomized trials in Sweden [24,25], Poland [26,27], Italy [28], Germany [29,30], and England [31,32].

Recently, to improve dissemination or *scaling out* prevention programs [33], an 11-lesson home-use DVD video series (SFP DVD) targeting families with children aged 7 to 17 years (SFP 7-17) was created for parents and youth to view together at home [34]. The 2-disk set, with an alternate Spanish audio track, is marketed at US $5 through a nonprofit foundation, with discounts for orders over 100 copies. In addition, the DVD is made available through internet streaming for families to view for US $5 per year [35]. The SFP DVD set includes handouts in both Spanish and English that can be printed off the disks themselves or downloaded free from the internet.

The SFP DVD was designed specifically to target key risk factors that contribute to youth substance use and teaches skills in bonding, setting boundaries, and parental monitoring. It included new material on healthy teen brain development, an animation of how neuroplasticity works through repeated and reinforced practice, tips for achieving school success, a kinesthetic tool for anger management, and brain scans from respected scientists showing the harms of alcohol and drugs. Mindfulness training was added to the SFP DVD in 2017 to help improve emotional regulation in parents and the youth who suffered adverse childhood experiences. It also teaches social skills that the youth need to resist negative peer influences, including how to say *no* to harmful things and still keep their friends.

Repetitive skill practice is an essential component of the SFP curriculum, and viewers are routinely invited to pause the DVD at key intervals and practice the skills they just learned. Each week, the parents and youth are given skills to practice and fun family goals to work toward. The SFP DVD thus offers a fairly complete package of family relationship tools that are targeted to reduce risk factors and increase protective factors related to substance use and delinquency.

Converting a group-based program to a video or DVD delivery format presents several logistic and methodological challenges. For instance, although the DVD provides greater program implementation fidelity, it does not involve group discussions that increase buy-in and foster problem solving. Similarly, the DVD does not include a family coach or facilitator to provide reinforcement and encourage practice—all considered essential active ingredients of the SFP program. Furthermore, the SFP DVD instructional modality departs from the traditional delivery methods that train the parents and youth separately for the first hour, followed by a joint skills practice session in the second hour. The ability for families to discuss their respective approaches to parenting and child management is another core active ingredient that contributes to the success of SFP.

Practicing skills, receiving immediate feedback, and learning about the different contexts of how skills can be used all foster behavioral improvements for both the parent and child. This raises the question of whether a joint parent-youth skills training program could work using video instruction with parents and the youth simultaneously engaging and observing the other during their instruction.

### Contrasting Delivery Modalities

This study compared outcomes from the standard group-based facilitator-led approach to various mixed uses of the SFP DVD using a noninferiority trial [36]. In contrast to the traditional *superiority* trials that use a placebo control condition (ie, minimal contact or an attention control), noninferiority trials are meant to compare interventions where the emphasis is on showing that a new treatment is *no worse than* a standard treatment, which in this case is the existing SF*P* 14-week group format. Noninferiority trials are usually implemented in the pharmaceutical industry where a drug treatment is contrasted with another that has already been shown to be effective; however, the second drug offers some amelioration of side effects or improved pharmacokinetics. The basic concept of this type of trial mirrors the present context where an intervention (standard SFP) has already been proven effective, but the second one (SFP DVD) offers some improvement in delivery and is more cost-efficient.

The focus here is whether the DVD can produce effects comparable with (or no worse than) the group-based program. As the DVD is more cost-effective, convenient to use, and has broader dissemination capability, it is valuable to test the performance of this delivery modality in comparison with the traditional group-based instructional methods. Before presenting the empirical findings, we first briefly discuss the theoretical rationale behind the SFP program, including discussion of the program’s active ingredients. We then describe the different settings where the SFP DVD has been implemented since its creation in 2012. We conclude by presenting empirical findings based on analyses contrasting the different delivery modalities.

### Theoretical Framework

The SFP is based on Family Systems intervention theories elaborated by Bowen [37], who observed in his clinical study that children’s problems were often rooted in the way parents dealt with or treated their children. The skills training format was influenced by the behavior change techniques of Skinner Operant Conditioning [38] and confirmed by Bandura Social Learning Theory, cognitive behavioral theories, and self-efficacy theories [39]. Teaching parents to use positive reinforcement (attention and praise) for wanted behaviors and ignoring unwanted behaviors were adapted from Patterson Cognitive Behavioral Change theories and skills training methods developed to reduce psychopathology in children and families [40,41]. These explanatory systems are then integrated with therapeutic skills–based techniques, including interpersonal and cognitive problem-solving methods [42] and relationship counseling strategies [43].

Patterson coercive family processes theory of delinquency and antisocial behavior [44] provides a social-interactional perspective highlighting the vital role family dynamics play in socializing the child for both pro- and antisocial behaviors. According to this perspective, various social-interactional contexts, mainly occurring in the home, can promote *coercive processes* that enmesh the parent and child in maladaptive patterns of behavior [45].

The cycle often begins with harsh and inconsistent discipline of a difficult child, followed by lax parental supervision and the inability of parents to socialize the child into adopting prosocial behavior. The child responds to the harsh environment by aggressively acting out in an effort to coerce the parent into submission, setting into motion a recurrent pattern of maladaptive parenting practices and hostile communication. To avoid further conflict, the parent often withdraws or, through frustration, chooses to disregard the child’s need for training rather than confronting additional hostility. These early patterns of problem behavior continue when the child enters school, where he or she transfers the negative behavioral interactions learned at home to their teachers and peers. This often contributes to rejection by the norm-following peers, leading the affected child to gravitate to deviant youth, who positively reinforce and shape their maladaptive behavior [46,47]. Their negative behavior increases levels of conflict in the home, which results in lower levels of parent-child involvement, which is related to poor parental monitoring and association with deviant peers [48].

Breaking this cycle requires training the parents to more effectively manage their child by spending quality time together doing fun activities, praising positive behaviors, using improved communication skills, setting expectations, and inculcating positive values. Parents also need to establish clear standards of behavior; give mild, consistent consequences for misbehavior; and monitor their child’s activities and peer relations [49,50].

The importance of parental influence in children’s behavior is supported by the statistically tested causal model using Structural Equations Modeling (SEM). This SEM-tested causal model found that 3 family cluster variables—family attachment or bonding, communication of positive family rules against substance use (boundaries), and parental supervision—were the most critical in protecting the youth from substance abuse [8]. These family-focused interventions proved to be particularly effective in reducing behavioral health disorders, drug use, and intermediate risk factors, such as conduct disorders, aggression, and family conflict. They also improved protective factors, such as social competencies, peer resistance skills, family and school bonding, school performance, and family organization and cohesion [17]. Similar SEMs have been tested for school failure, delinquency, teen pregnancy, and alcohol and drug use with similar results [51].

### Active Ingredients

SFP lessons begin with skills to reduce hostility and create warm, loving relationships between the parents and child. The parents learn and practice nurturing skills, including one-on-one playtime (allowing the child to choose the activity), giving positive attention through daily looking for and complimenting the good and avoiding criticism; engaging in pleasant communication, including active listening and validating each other; eliminating communication boulders (eg, yelling, swearing, and sarcasm); and learning to have fun weekly family meetings.

Later, skills involving boundary setting are introduced, with each family making their own personal family rules with input from their children. A reward system is set up for following house rules. This lays the foundation to introduce the skill of positive discipline that involves teaching and rewarding the behaviors parents want, with each family (parents and their children) deciding on fair, mild negative consequences that will be delivered calmly and consistently for misbehavior.

The lesson material on problem solving, stress reduction, thinking ahead to stay out of trouble, anger management, school success, substance use education, and parental monitoring follows. Finally, family traditions, values, and community service are introduced, with encouragement for children to become a positive agent of change.

Skills training for children parallels the parent lessons, with additional emphasis on emotional regulation and self-management skills, peer drug refusal and social skills, and the importance of choosing prosocial friends.

The core active ingredients of SFP can be administered in varying dosages, allowing a service provider (eg, family service agency) to choose the level of intervention according to the risk levels of individual families. Lower dosage versions, including the SFP DVD and 10-session group classes, are used for *universal* prevention with low-risk families. Higher dosages are used for *selective* and *indicated* prevention and treatment in high-risk families with at-risk youth, delinquents on probation, or child maltreatment cases, with trained family coaches often delivering the SFP DVD in-home to those most at risk.

## Methods

### Strengthening Families DVD Program Delivery Methods

Although the SFP DVD was created primarily for home use, various implementation strategies have evolved to include creative, off-label ways to incorporate the DVD. Table 1 shows the different settings where the SFP DVD has been implemented. Of these, 3 strategies involving the SFP DVD are the focus of the present analyses: (1) home-use with *no family coach*, (2) viewing the SFP DVD as part of a family discussion group, and (3) shorter 10-week SFP 7-17 group classes for parents and the youth that also included DVD video clips.

Although worthy of mention, the remaining venues listed in the table are not examined in the effect size (ES) comparison because they differed in the assessment protocol (using an abbreviated survey) and study design (true pre-posttest rather than a retrospective design) or delivery method (to parents via middle school health class assignments that required viewing only 3 lessons—the Introduction and lessons 8 and 9). The 3 primary settings examined in this paper (DVD at home, DVD family discussion group, and parent-youth classes plus DVD clips) all used a quasi-experimental design with retrospective pre-post reporting and are briefly described here.

**Table 1:** A total of 5 settings that utilized the Strengthening Families Program DVD for family-based prevention.

Setting, Study Method (year)		Study design	Sample number^a^	Retention^b^	Recruitment method	Implementation information
**Home-use DVD**						
	Families viewed DVD at home with no coach—2 rounds (2012)	RPP^c^	55	27 (23); 34 (32)	Letters mailed by school to parents asking to view	SFP^d^ DVD mailed to families in the Salt Lake City School District who volunteered to watch the DVD and take a Web-based survey in exchange for donated prizes; parents took a standard SFP survey via SurveyMonkey; challenges^e^—finding volunteers to watch the DVD lessons
	Asian Indian families viewed DVD at home—no coach (2013)	RPP	26	28 (26)	Flyers in Indian stores and temples	Asian Indian families watched the DVD at home and took a paper-and-pencil survey; contacted via flyers at Indian grocery stores; challenges—parents not home, getting parent consent forms signed when delivering DVD to each family
**DVD family discussion group**						
	Open Classroom Elementary School—Library (2013)	RPP	9	9 (9)	Parent Teacher Association newsletter and flyers at school	School counselor invited parents to attend; 9 finished a 10-week class and took a paper-and-pencil survey; parents eager to come and learn
	Road Home homeless shelter (2013)	RPP	11	11 (11)	Notices posted in Road Home shelter	11 single mothers and children finished the course; others found housing and left before the course ended; additional nutrition information included
	Washington County Youth Crisis Center (2014)	RPP	9	9 (9)	Flyers posted in schools	Held in Hurricane, UT^f^; money for dinners from Youth Crisis food budget; gave staff time off during workday to teach SFP class at night
	The Journey (2015)	RPP	16	16 (10)	Youth in detention; parents came to Friday night class	Parents and their delinquent child who was in custody watched DVD as a group; the youth returned home for the weekend, and then received visit at home from an SFP coach to practice new skills
**Strengthening Family Program class plus DVD clips^g^**						
	UT-Salt Lake City group classes, 2 sessions—spring and fall (2013)	RPP	29	8 (8)	Flyers and phone calls	Program taught by college student interns; food and evaluation funded by University of Utah grant
	UT-UVU^h^ intern classes (2013)	RPP	115	19 (13), 6 (6); 5 (5), 27 (27); 8 (8); 8 (8); 10 (10)	Middle school counselors advised parents to attend	SFP for families with children aged 7-17 years classes taught 3 times a year by UVU student interns at multiple sites; schools asked families to attend because of child behavior issues; food donated by a local church
	UT-Payson City (2014)	RPP	11	11 (11)	Flyers posted at schools	Classes funded by the city council; participants self-selected to attend; had a waiting list of parents to attend
	TX^i^-Conroe—Spanish (2016)	RPP	24	24 (13)	Church flyers	All Spanish speaking; held at a church; the pastor wanted all families to attend at once; hired extra coaches to teach; church members made food
	NV^j^-Reno—Boys and Girls Club^g^ (year 1; 2016)	TP^k^	32	—^l^	Flyers at Boys and Girls Club	Taught at multiple sites; used a shorter 48-question survey that was mailed to agencies and analyzed by an independent evaluator
	NV-Reno—Boys and Girls Club^g^ (year 2; 2018)	TP	98	—	Flyers at Boys and Girls Club	Waiting list for families to attend; surveys analyzed through Gravic Remark system
**View DVD in-home with family coach**						
	North Carolina agencies (year 1; 2017)	TP	56	—	Family coaches taught SFP via DVD, in-home training, and counseling program	Year 1: SFP taught as part of in-home intensive therapy program to low-functioning families in 7 behavioral health agencies in North Carolina; Brilliance Analytics pre- and post surveys mailed to clients with self-addressed stamped envelope for return mail
	North Carolina agencies (year 2; 2018)	RPP	47	—	Same as above	Year 2: same survey questions in a new format; scanned into a computer and analyzed through Gravic Remark software system
**View DVD in-home as school health class assignment**						
	Salt Lake City School District^m^ (2012)	Mixed	364	—	Middle school health teachers gave parents SFP DVDs with an assignment to watch 3 lessons with their child and fill in and return home worksheets	Students received the DVD in the 7th grade health class; mandatory homework to watch 3 lessons with parents (Introduction and lessons 8 and 9) and take a brief pre-post survey; a year later, those students took the Student Health and Risk Prevention^n^ survey in the 8th grade; students who had an assignment to view the DVD said that parents talked to them more about alcohol, tobacco, and other drugs and checked up on them more than students who did not receive the DVD; 8th grade binge drinking rates also declined by 50% in the school district; Bach Harrison conducted analyses

^a^Number of families initially enrolled.

^b^Numbers in parentheses indicate the final tally of parents who provided a pre- and posttest and completed the course.

^c^RPP: retrospective pre-posttest.

^d^SFP: Strengthening Families Program.

^e^Challenges depended on experience-level of family coaches and site coordinator; first year challenges (except for church group and court-ordered families) were mainly getting enough people to attend.

^f^UT: Utah.

^g^SFP 7-17 for families with children aged 7 to 17 years; group classes are 10 weeks long versus 14 weeks for regular SFP group-based, facilitator-led classes. Facilitators showed clips of the DVD during class, showing examples of skills they were teaching.

^h^UVU: Utah Valley University.

^i^TX: Texas.

^j^NV: Nevada.

^k^TP: true pre- or posttest; studies using TP or mixed design were not used in the effect size analyses.

^l^Not applicable.

^m^Analyzed by an independent evaluator.

^n^The Student Health and Risk Prevention survey—a biannual statewide survey given to students during school in 6th, 8th, 10th, and 12th grades—used items from Monitoring the Futures survey.

#### Home-Use DVD

The first efficacy trial of the home-use DVD required the families with children aged 7 to 17 years to view the 11 SFP DVD lessons and participate in a confidential Web-based survey in exchange for entering into a drawing for valuable prizes donated by local merchants. A total of 61 families with children from 3rd to 12th grade volunteered to watch the 11 DVD lessons. The DVD was mailed to the families to view at home together. Of them, 55 families completed the DVD lessons and took the regular SFP retrospective pre-post survey on the Web.

The results of the survey were compared with a shorter, updated 10-week version of the group class that included video clips taken from the SFP DVD. This was titled *SFP 7-17*, and the classes were taught in the evenings at 2 Salt Lake City elementary schools by the University of Utah graduate student interns. A randomized block design was used with all 6th and 7th grade schools. Schools characterized by few risk factors for substance abuse were put into a group and those with multiple risk factors were placed in another group. From these, we randomly selected a school that was a relatively high-performing school, whereas the other was a Title 1 school, with 90% of the students receiving free or reduced-price lunch. A majority of the Title 1 school parents spoke Spanish, so the parent training classes were taught in 2 groups—1 in English and 1 in Spanish. The youth preferred to be taught in English, and the family practice session was taught in both languages with the help of translators. At the end of the 10-week class, parents in both schools completed the regular SFP paper-and-pencil retrospective pre-post survey (in English or Spanish, depending on preference).

A second trial of the home-use DVD involved Asian Indian families who were experiencing acculturative stress arising from differences between their strict, authoritarian rearing conditions in India and their US-born children growing up in a more liberal, westernized society. Inclusion criteria required that parents (1) were born and raised in India and had children aged between 7 and 17 years who were born and raised in the United States and (2) agreed to watch the 11 DVD lessons and pause and practice the skills where indicated. A total of 28 families volunteered, and 26 finished the DVD lessons and took a paper-and-pencil version of the retrospective pre-post survey. The results of the Asian Indian survey were compared with the SFP 7-17 10-week group class version, with video clips taken from the SFP DVD. The classes were held in the evenings at local middle schools and taught by the Utah Valley University sociology student interns.

#### DVD With Family Discussion Group

Given drastic budget cuts, family service agencies began to use the SFP DVD as a cost-effective means of delivering SFP skills. Instead of hiring 6 staff members to teach the parent, teen, and child lessons, they gave 2 regular staff people time off during the week and had them come in 1 evening a week to act as a family coach as they led the SFP DVD family discussion group. They paused the DVD at set intervals, asked discussion questions, and had the parents and youth do *practice walk-throughs* of the skills they were viewing. We have included 4 settings that involved the SFP DVD shown in a group format: an open-education learning environment that took place in an elementary school, a homeless shelter, a crisis center, and a mixed residential and outpatient youth detention facility. In all cases, several families watched the DVD as 2 facilitators paused it where indicated, asked discussion questions from the SFP DVD discussion guide, and led the parents and youth in joint skills practice. In the residential setting, the youth in custody at the facility were taught skills from the DVD during the week. On Friday evenings, their parents visited the facility, watched the DVD in a group setting with their youth, and practiced the skills with them. At the end of the 11 lessons, all parents in their respective groups took the regular paper-and-pencil retrospective pre-post survey.

#### Families Participated in Strengthening Families Program 7 to 17 Group Classes and Viewed DVD Clips

The 10-week SFP 7-17 group class curriculum teaches the same skills as the regular SF*P* 14-week lessons, with a slight variation in the order they are presented. Additional DVD course material was added on brain development; parental involvement via pleasant personal conferences; apologies and forgiveness; anger management; harms of alcohol, tobacco, and other drugs; and mindfulness to stop automatic negative thoughts. The SFP 7-17 lessons, which follow the same order as the SFP DVD, use video clips from the DVD to demonstrate the skills being taught.

Since 2013, classes have been offered in the evenings in various community settings, including family service agencies, churches, and schools. SFP 7-17 includes separate 1-hour classes for parents, teens, and children plus a joint family practice session in the second hour. The sessions last 10 or 11 weeks, compared with the regular 14-week SFP group class.

#### Strengthening Families Program Group Norms

The group norms chosen for the noninferiority trial comparison came from a database of over 6000 families who had previously taken the SF*P* 14-week group classes and filled out a retrospective pre-posttest. A sample of 473 representative families were randomly chosen from a variety of sites based on similar demographics (ages of the children) and the proximity of the classes to the dates corresponding to the DVD implementation. Owing to the diversity of sites where the group norm families attended the classes, it is not possible to determine implementation issues at the sites. However, retention at the group norm sites varied between 80% and 95% across the 14 weeks, depending on the teachers’ experience levels and buy-in from program directors.

### Measures

The SFP assessment protocol uses reliable scales that, in the interest of time and reducing participant burden, are abridged versions of psychometrically sound assessments. Estimates of internal consistency presented here are based on the group norms sample with the exception of the covert aggression scale, which is based on a larger study conducted in Ireland. A total of 5 multi-item scales assess parenting-related skills including parental involvement (eg, “I talk to my youth about his or her plans for the next day or week”; alpha=.75), parental supervision (eg, “I know where my child is and who he/she is with”; alpha=.70), parenting efficacy (eg, “I handle stress well”; alpha=.75), positive parenting (eg, “I praise my child when he/she behaves well”; alpha=.79), and parenting skills (eg, “I use physical punishment when my child will not do what I ask”; alpha=.64). Items for the parenting skills, parental supervision, and positive parenting scales were taken from the *Kumpfer SFP Skills* instrument [52], and parental involvement items were taken from the *Alabama Parenting Scale* (APS) [53,54]. Recent psychometric evidence confirms the reliability of shortened scales from the APS [55,56]. In addition, 4 abridged scales were taken from the *Moos Family Environment Scale* [57,58] to assess family cohesion (eg, “I enjoy spending time with my child”; alpha=.75), family communication (eg, “We hold a family meeting weekly”; alpha=.69), family conflict (eg, “Our family argues a lot with each other”; alpha=.87), and family organization (eg, “We go over schedules, chores, and rules to get better organized”; alpha=.71).

Items assessing cognitive, affective, and behavioral facets of depression were taken from a survey instrument used to evaluate the *Good Behavior Game*, a school-based intervention to reduce aggression, delinquency, and drug use [59]. The items were originally culled from the *Child Depression Inventory* [60,61] and the *Child Behavior Checklist* [62,63]. Parents rated their children’s mood and emotional tone with 6 items (eg, “My child looks sad or down”; alpha=.64), making sure to simplify the wording for families with language or education barriers. A 6-item scale was used to assess covert aggression (eg, skipping school or breaking rules; alpha=.69), and separately, another 6-item scale assessed overt aggression (eg, hitting or fighting; alpha=.75). All scales were adapted from the Parent Observation of Child Adaptation (POCA) scale [64]. The POCA assesses how the child conforms to the family social world (ie, their aggressive and disruptive behavior) and is a modification of the Teacher Observation of Classroom Adaptation–Revised questionnaire [65] assessing a child’s performance on core classroom tasks (ie, accepting authority, social participation, and concentration) and their social adaptational status. The teacher rating instrument was developed originally as part of the Woodlawn, Chicago, early behavior management intervention study [66] and then later used in evaluating the *Good Behavior Game* intervention [64,67].

A 9-item scale assessing social behavior (ie, cooperation, assertion, responsibility, and self-control; eg, “My child plays well with other children”; alpha=.79) was taken from the Social Skills Rating Scales [68,69]. We used a 12-item scale to assess family strengths and resilience (eg, “We show that we care for each other in our family”; alpha=.90), developed as an abridged version of a performance checklist used in child abuse and neglect cases [70,71]. Parents were also asked to evaluate their child’s past month use of alcohol, cigarettes, marijuana, and prescription medication drugs both before (eg, “In the 30 days before the SFP class, how many times do you think your child used the following”) and again after the exposure to the SFP program (now). These scales were taken from nationally representative epidemiological surveys targeting the youth [72] and are based on counts. For all of the scales, the study calculated average scores for the parent, child, and family outcomes.

### Analysis Methods

A statistical analysis was performed comparing the ES of the 3 SFP DVD conditions to the group norms. The statistician compared site characteristics using chi-square tests for categorical and analysis of variance (ANOVA) for continuous measures. The ES estimates (partial eta-square) for the outcomes were calculated using a within-subjects repeated measures of ANOVA (the interaction of time by condition was tested) [73-75]. This is an appropriate choice of ES when the designs being compared are similar. To avoid issues with power, we bundled several of the *off-label* settings into 3 distinct groups based on the delivery method (group 1 delivered the DVD entirely at home, group 2 included families that viewed the DVD as part of DVD family discussion groups, and group 3 attended SFP 7-17 group classes and viewed DVD video clips).

He then compared the resulting 3 conditions to the traditional facilitator-led SF*P* 14-week group format (group norms). Following standards for noninferiority trials [36,76], he used an equivalence margin based on the null hypothesis, stating that the DVD conditions would be no worse (or better) than 10% difference in ES estimate compared with the group norm condition. This level for the margin of equivalence was set because we expected all forms of the treatments to be at least similar.

## Results

### Site Comparisons

A total of 711 participants were divided among the 4 conditions: group norms (473), home-use DVD (81), family discussion group (39), and SFP 7-17 class with DVD clips (118). ANOVA was used to compare the 4 different conditions on demographic factors, including the age of the parent and child, and chi-square tests were used to compare the conditions on race and family status (eg, single parent, 2 parents, joint/shared custody, foster care, relatives, and other). Percentages below for race categories across all 4 comparison conditions were based on the 677 out of 711 participants who marked the “race” category. They included African American 21.1% (143/677), Asian 12.6% (85/677), White 32.8% (222/677), Hispanic 26.6% (180/677), and a mixed group comprised of Native American, Hawaiian, Pacific Islander, and Alaskan natives 6.9% (47/677).

Significant condition differences were observed for race (X^2^_12_=219.0; *P*<.001). In the group norm classes the greatest percent were African American families 31.3% (142/453) compared to Hispanic families 26.9% (122/453, White families 21.9% (99/453), Asian families 12.4% (56/453), and the mixed-race group 7.5% (34/453). In the home use DVD group Whites were the highest at 58% (47/81) followed by Asian 34.6% (28/81), Hispanic 7.4% (6/81), with African American and mixed race groups not represented. In the DVD family discussion group Whites were 39.3% (11/28), mixed race 32.1% (9/28), Hispanic 25% (7/28), and African American 3.6% (1/28). Asian was not represented. In the SFP 7-17 classes with DVD Clips Whites were 56.5% (65/115), Hispanics were 39.1% (45/115), mixed race 3.5% (4/115), and Asian 0.9% (1/115). African American was not represented. There were no significant differences in the gender of the parent filling out the survey (X^2^_3_=3.84; *P*=.28) or the gender of the target child (X^2^_3_=6.47; *P*=.09).

Comparison of family status was significant (X^2^_15_=25.42; *P*=.045). There were 242 single parents in the sample. 78.5% (190/242) were in the group norms; 10.7% (26/242) were in the SFP 7-17 classroom with DVD clips condition; 8.3% (20/242) were in the home-use DVD condition; and 2.5% (6/242) were in the DVD family discussion group condition. There were also no foster care children in any condition other than group norms; but that imbalance may reflect the recruitment strategies more than anything.

Parents were much younger in the family discussion group (mean age 26.8 years), compared with the remaining groups (*F*_3,610_=8.52; *P*<.001; mean age 40.7, 39.3, and 39.5 years for group norms, home-use DVD, and classroom with DVD clips, respectively). Children were also significantly younger in the family discussion group (*F*_3,636_=18.78; *P*<.001; mean age 10.89 years), compared with the other 3 conditions (mean age 13.74, 13.15, and 12.77 years for group norms, home-use DVD, and SFP 7-17 classroom with DVD clips, respectively).

A comparison of income across the 4 conditions was found to be significant after conducting the nonparametric Kruskal-Wallis test (ie, a 1-way ANOVA, H_3_=65.575; *P*<.001). Home-use DVD families reported the highest average income (US $51,220, SD US $57,872) compared with SFP 7-17 classroom with DVD clips (US $44,876, SD US $38,790), family DVD discussion group (US $42,342, SD US $24,778), and group norms (US $27,878, SD US $25,911).

### Effect Size Estimate Comparisons

Multimedia Appendix 1 shows the unadjusted ES comparisons for group norms versus the 3 DVD conditions. Notably, none of the group×time interactions were significant. Of the 15 comparisons for the SFP outcomes, 13 favored the DVD with larger ES estimates.

As seen in Multimedia Appendix 1, the average ES difference between group norms and the home-use DVD condition was 0.13. The margin of equivalence favored the home-use DVD with the ES at least 28% larger by comparison (family communication favored the group norms). The largest ES overall for the home-use DVD condition was observed for family strengths/resilience (0.76 vs 0.65 for home-use DVD and group norms, respectively) followed by family organization (0.73 vs 0.64). Interestingly, the smallest ES was for youth alcohol and drug use (0.20 for home-use DVD and 0.01 for group norms), which may reflect the low perceived levels of child drug use in this sample.

ES comparisons for the other SFP DVD use conditions were in some cases somewhat larger in magnitude, compared with the home-use versus group norms comparison. For instance, the average ES difference for the DVD family discussion group compared with the group norms was 0.16, and the average margin of equivalence was 31% larger for the DVD family discussion group condition, compared with the group norms. Individual ES comparisons showed the largest ES for the DVD family discussion group condition was for family strengths/resilience (0.79 vs 0.65 for group norms), and this effect was also larger than the other conditions as well (0.76 and 0.70 for home-use DVD and SFP 7-17 classroom plus DVD Clips, respectively). Social behavior (0.74), parenting efficacy (0.73), family communication (0.72), and family organization (0.72) also had relatively large ES compared with group norms (0.34, 0.56, 0.66, and 0.64, respectively).

The same comparison for the SFP 7-17 10-week classroom version that included DVD clips indicated an average ES difference of 0.09 and an average margin of equivalence of 23%. The largest magnitude of individual ES was for family organization (0.72) and communication (0.71), both of which were larger in magnitude compared with the group norms (0.64 and 0.66, respectively) and the home-use DVD.

### Adjusted Effect Size Analyses

It is conceivable that site-specific variability may influence scores within each condition and thus contribute to ES differences. This variability can arise from differences in the composition of the participants at each site. To test the effect of intersubject variability, we computed adjusted ES, modeling the influence of demographic measures (eg, adult and child gender, age, and race and family income). Multimedia Appendix 2 shows the results of the ES comparisons with the adjustments conducted with forward inclusion and modeling first-order interactions. As depicted, there was some shrinkage in the ES as the additional measures accounted for demographic variance. However, the overall consistency of the findings did not change, reinforcing the superior effects obtained with the DVD conditions.

## Discussion

### Principal Findings

This study provides initial evidence that the SFP DVD provides a useful surrogate for the traditional group format that uses a facilitator, which was how SFP was initially developed. For a variety of reasons, many families find it difficult to maintain their attendance at the various sites where SFP is traditionally delivered. This has been a consistent and well-noted issue associated with offering any parenting skills training program [77,78]. Parents are busy with work, caring for their children, and handling chores crucial to their survival. Many families, faced with hectic schedules, afterschool activities, and other competing interests, find it difficult to attend a 2-hour family-based skills training for 10 to 14 weeks that requires transportation to and from the facility. With the advent of the SFP DVD, parents and their children can have access to the program content, modified slightly, in an alternative setting—one of which does not require attendance per se in a fixed edifice or a labor-intensive group delivery format. Furthermore, they can review the SFP skills as often as necessary at home.

The results of the noninferiority trial show that the home-use DVD was superior to the group norms in all but 2 of the 15 comparisons. The superiority exceeded the benchmark of 10% set a priori before the trial commenced. The 2 outcomes that failed to exceed the group norms were family cohesion (which had identical ES) and communication (change in ES=0.03). Both of the other 2 DVD conditions had ES larger in magnitude for these 2 outcomes compared with the group norms. In this respect, we were able to demonstrate that using the same experimental design and generating partial eta-square statistics to create a common metric for study comparison, the DVD conditions produced superior effects to the traditional group-based format.

Although we computed ES based on a within-subject design, intersubject differences based on sample composition can also influence ES computations. This arises because the computation of the ES in an ANOVA framework utilizes the sums of squares, which is inextricably tied to the raw mean scores. Taking this into consideration, we computed adjusted ES for each condition, controlling for demographic characteristics of the sample. The adjusted ES left the same impression as the unadjusted, that is, all the conditions with the DVD outperformed the group norms. The margin of equivalence favored the home-use DVD and the DVD family discussion group at relatively the same magnitude as the unadjusted calculations.

Comparatively speaking, although most of the DVD conditions outshone the group norms, there were several SFP outcomes that produced less than optimal ESs. This observation is guided by general standards for Cohen *d* and takes into consideration how the partial eta-square converts to Cohen *d* [79]. The benchmark numbers suggest that an ES equivalent to *d*=0.2 is small, *d*=0.5 is moderate, and *d*=0.8 is considered large [74]. If the ES is 0.5, as it was in many cases here, the families improved a half standard deviation over time. Smaller effects (eg, *d*=0.2-0.4) mean that the family did not improve as much (with ratings obtained from the parent’s perspective only). In summary, across the different comparisons, there were several ESs that were relatively small, including family conflict, depression, and covert and overt aggression. A pressing question, then, is why these ESs are smaller in magnitude and tied to this concern, what contributes to the differences in program outcomes?

The strength of SFP is its focus on improving parenting skills (which increased to a considerable degree) and its carryover effect on youth behaviors. The inclusion of aggression and depression scales, although not primary outcomes, are intended to foreshadow what may happen when family dynamics improve following program exposure. This view is consistent with a developmental cascade model suggesting that behaviors in a domain can sequentially influence behaviors in a different domain through spreading activation effects and because skills for both the parent and child invariably emerge from a common foundation [80-82]. Thus, activation of negative behaviors at home can *spread* to school or adversely affect peer relations both within and across time, setting into motion developmental pathways that foster maladjustment in multiple domains. The risk-factor model and social transactional perspective underlying SFP integrates this approach, suggesting that coercion and poor parenting skills in an area (eg, boundary setting) can *cascade* and influence other behaviors (eg, family bonding), upsetting the balance of family dynamics. Improvements in the way parents discipline or set boundaries, for instance, can have repercussions on family bonding or monitoring in a positive way by bringing the family closer, improving parent-child communication, and lessening the impact of negative behaviors.

### Accounting for Delivery Format Differences

There were other instances where the home-use DVD did not produce larger ES compared with the group norms; and the SFP 7-17 groups classes with video clips out performed them all. It is possible that without a family coach or facilitator to monitor, encourage, and correct their skill practice, families deeply embroiled in conflict have more trouble changing communication patterns, especially in a short amount of time. For these families, certain behaviors may be intransigent, and efforts to change these highlight the benefits of having a family coach who can provide skill reinforcement to instigate behavior change. Yet, the majority of comparisons reinforced that ESs for the DVD conditions surpassed the group norms.

The superiority of DVD outcomes may be affected by the enriched content that was added or demographic differences, as parents in the home-use DVD condition had higher income levels and the functional ability to gather their children to watch the DVD and practice the skills at home. Higher levels of functionality can include more time spent bonding and watching the SFP videos and discussing their content. In addition, watching the DVD at home allows families to pause the instruction, practice skills, and review sessions multiple times at their own convenience and pace. This provides a customized delivery not available with classroom-based instruction, where pace is dictated by the facilitator and the group dynamic. The ability to customize presentation could help offset 2 recurring problems in family-based prevention, including attrition and engagement [83].

When adjusted, the ES comparison indicated some decrement in program outcomes. Clearly, factors related to the demographic composition of these families had an influence on their mean scores to the extent that there was some small shrinkage in the ES as seen in Multimedia Appendix 1. Overall, the DVD conditions had more families improving on the 15 outcomes.

### Limitations

There are several limitations to this study worth noting. First, all of the studies included in the ES comparisons relied on retrospective pre-post reporting, thus eliminating any passage of time between assessments. There are advantages and disadvantages to this type of reporting method because it relies on *retrospective* recall of skills and behaviors that can be tarnished by memory. However, in a design that uses a true pretest separated in time from the posttest, parents are prone at baseline to evaluate themselves in a glowing light and consider themselves more effectively skilled. This personal evaluation changes dramatically when the same parent sits through the SFP lessons, learns new skills, and realizes they had less than optimal parenting skills at the beginning. As a result, the retrospective pre-post format allows parents to answer how they are currently parenting at the conclusion of the study and then *reflect* back on their earlier parenting skills and evaluate the improvements made following exposure to course content. This is one of the strengths of retrospective pre-post techniques [84], as it helps participants to generate an internal *standard of comparison* by asking them to address their parenting skills looking back over a few months’ time and compare them to their parenting skills after program exposure. This technique provides an anchor for the parent and avoids any *response-shift bias* as a threat to internal validity, which may provide a more accurate assessment [85].

In comparing the group norms to the DVD conditions, we set the margin of equivalence at 10%, which is an arbitrary benchmark value. However, setting an even stricter level of scrutiny for the null, for example, 20%, would still have produced evidence of noninferiority for the DVD conditions. Despite noninferiority trials having their limitations [86], they can still be used, as is the current case, for illustrative purposes to show that a novel implementation strategy is no *worse* than an effective treatment control. Future studies may want to rely on RCT designs to strengthen causal inferences about program effects.

We also did not control for numerous factors that may contribute to the differences in study outcomes, including family risk factors, compliance with the study protocol, attrition, and measurement error. Facilitators in the group norms and DVD conditions can introduce variance into the equation, affecting program adoption and fidelity in ways that we did not account. Moreover, the sampling mechanisms were nonrandom, and this could lead to bias in the ES estimates. Given that randomization was not used in any of the trials, intent-to-treat analyses were essentially moot.

### Conclusions

Even with these noted limitations, there is a tremendous need to train parents and their children with appropriate evidence-based skills to avoid alcohol and drug use, as well as other delinquent behaviors. On-site classes are the standard effective mode of instruction; but they can never meet the rising demand because of higher costs and reduced prevention budgets. Marrying technology with primary prevention appears to be the most viable way to offer skills training to enough parents and their youth to make an appreciable difference in decreasing delinquency and youth alcohol and drug use. The SFP DVD offers an engaging and inexpensive way to bring evidence-based programs to scale to reduce adolescent behavioral problems and social costs.

## Appendix

Multimedia Appendix 1 Table 2. Comparison of Strengthening Family Program (SFP) group norms with 3 SFP DVD conditions.

Multimedia Appendix 2 Table S1. Comparison of unadjusted and adjusted effect sizes for Strengthening Families Program (SFP) Noninferiority Trial.

## Abbreviations

ANOVA: analysis of variance
APS: Alabama Parenting Scale
ES: effect size
POCA: Parent Observation of Child Adaptation
RCT: randomized controlled trial
SEM: Structural Equations Modeling
SFP: Strengthening Families Program
SFP 7-17: Strengthening Families Program for families with children aged 7 to 17 years

## References

[1] Tapert SF, Granholm E, Leedy NG, Brown SA (2002). Substance use and withdrawal: neuropsychological functioning over 8 years in youth. J Int Neuropsychol Soc.

[2] Clark D, Thatcher D, Tapert S (2008). Alcohol, psychological dysregulation, and adolescent brain development. Alcohol Clin Exp Res.

[3] Chambers RA, Taylor JR, Potenza MN (2003). Developmental neurocircuitry of motivation in adolescence: a critical period of addiction vulnerability. Am J Psychiatry.

[4] Johnston LD, Miech RA, O’Malley PM, Bachman JG, Schulenberg JE, Patrick ME (2018). Monitoring the Future.

[5] Kumpfer KL, Hansen WB, Scheier LM, Hansen WB (2014). Family-based prevention programs. Parenting and Teen Drug Use: The Most Recent Findings From Research, Prevention, and Treatment.

[6] (2010). United Nations Office of Drugs and Crime.

[7] Van Ryzin MJ, Kumpfer KL, Fosco G, Greenberg M (2016). Family-Based Prevention Programs for Children and Adolescents: Theory, Research, and Large-Scale Dissemination.

[8] Kumpfer KL, Scheier LM, Brown J (2018). Strategies to avoid replication failure with evidence-based prevention interventions: case examples from the strengthening families program. Eval Health Prof.

[9] Kumpfer KL, Turner CW (1990). The social ecology model of adolescent substance abuse: implications for prevention. Int J Addict.

[10] Kumpfer KL, Johnson JH (1999). Factors and processes contributing to resilience: the resilience framework. Resilience and Development: Positive Life Adaptations.

[11] Biglan A, Flay BR, Embry DD, Sandler IN (2012). The critical role of nurturing environments for promoting human well-being. Am Psychol.

[12] Patterson GR, Forgatch MS, Degarmo DS (2010). Cascading effects following intervention. Dev Psychopathol.

[13] Kumpfer K, DeMarsh J (1985). Prevention of chemical dependency in children of alcohol and drug abusers. NIDA Notes.

[14] Demarsh J, Kumpfer KL (1986). Family-oriented interventions for the prevention of chemical dependency in children and adolescents. J Child Contemp Soc.

[15] Kumpfer KL, Whiteside HO, Greene JA, Allen KC (2010). Effectiveness outcomes of four age versions of the Strengthening Families Program in statewide field sites. Group Dyn.

[16] Kumpfer KL, Magalhães C, Whiteside H, Xie J, Van Ryzin MJ, Kumpfer KL, Fosco GM, Greenberg MT (2016). Strengthening families for middle/late childhood. Family-Based Prevention Programs for Children and Adolescents: Theory, Research, and Large-Scale Dissemination.

[17] Gottfredson D, Kumpfer K, Polizzi-Fox D, Wilson D, Puryear V, Beatty P, Vilmenay M (2006). The Strengthening Washington DC Families project: a randomized effectiveness trial of family-based prevention. Prev Sci.

[18] Kumpfer KL, Alvarado R, Tait C, Turner C (2002). Effectiveness of school-based family and children's skills training for substance abuse prevention among 6-8-year-old rural children. Psychol Addict Behav.

[19] Marek LI, Brock DP, Sullivan R (2006). Cultural adaptations to a family life skills program: implementation in rural appalachia. J Prim Prev.

[20] Kumpfer KL, Pinyuchon M, Teixeira de Melo A, Whiteside HO (2008). Cultural adaptation process for international dissemination of the strengthening families program. Eval Health Prof.

[21] Kumpfer KL, Alvarado R, Smith P, Bellamy N (2002). Cultural sensitivity and adaptation in family-based prevention interventions. Prev Sci.

[22] Kumpfer KL, Xie J, O’Driscoll R (2012). Effectiveness of a culturally adapted strengthening families program 12–16 years for high-risk Irish families. Child Youth Care Forum.

[23] Puffer ES, Annan J, Sim AL, Salhi C, Betancourt TS (2017). The impact of a family skills training intervention among Burmese migrant families in Thailand: a randomized controlled trial. PLoS One.

[24] Skärstrand E, Larsson J, Andréasson S (2008). Cultural adaptation of the Strengthening Families Programme to a Swedish setting. Health Educ.

[25] Skärstrand E, Sundell K, Andréasson S (2014). Evaluation of a Swedish version of the Strengthening Families Programme. Eur J Public Health.

[26] Foxcroft DR, Callen H, Davies EL, Okulicz-Kozaryn K (2017). Effectiveness of the strengthening families programme 10-14 in Poland: cluster randomized controlled trial. Eur J Public Health.

[27] Okulicz-Kozaryn K, Foxcroft DR (2012). Effectiveness of the Strengthening Families Programme 10-14 in Poland for the prevention of alcohol and drug misuse: protocol for a randomized controlled trial. BMC Public Health.

[28] Ortega E, Giannotta F, Latina D, Ciairano S (2012). Cultural adaptation of the Strengthening Families program 10–14 to Italian families. Child Youth Care Forum.

[29] Baldus C, Thomsen M, Sack P, Bröning S, Arnaud N, Daubmann A, Thomasius R (2016). Evaluation of a German version of the Strengthening Families Programme 10-14: a randomised controlled trial. Eur J Public Health.

[30] Bröning S, Baldus C, Thomsen M, Sack P, Arnaud N, Thomasius R (2017). Children with elevated psychosocial risk load benefit most from a family-based preventive intervention: exploratory differential analyses from the German 'Strengthening families program 10-14' adaptation trial. Prev Sci.

[31] Kumpfer KL, Molgaard V, Spoth R, Peters RD, McMahon RJ (1996). The Strengthening Families program for the prevention of delinquency and drug use. Preventing Childhood Disorders, Substance Abuse, and Delinquency.

[32] Spoth R, Redmond C, Mason WA, Schainker L, Borduin L, Scheier LM (2015). Research on the Strengthening Families program for parents and youth ages 10-14: Long-term effects, mechanisms, translation to public health, PROSPER partnership scale up. Handbook of Adolescent Drug Use Prevention: Research, Intervention Strategies, and Practice.

[33] Aarons GA, Sklar M, Mustanski B, Benbow N, Brown CH (2017). 'Scaling-out' evidence-based interventions to new populations or new health care delivery systems. Implement Sci.

[34] Kumpfer K, Brown J (2012). New Way to Reach Parents: A SFP DVD.

[35] Strengthening Families Program.

[36] Hahn S (2012). Understanding noninferiority trials. Korean J Pediatr.

[37] Bowen M (1974). Alcoholism as viewed through family systems theory and family psychotherapy. Ann N Y Acad Sci.

[38] Skinner BF (1938). The Behavior of Organisms: An Experimental Analysis.

[39] Bandura A (1986). Social Foundations of Thought and Action: A Social Cognitive Theory.

[40] Patterson GR, DeGarmo D, Forgatch MS (2004). Systematic changes in families following prevention trials. J Abnorm Child Psychol.

[41] Patterson GR, Bank L, Gunnar MR, Thelen E (1989). Some amplifying mechanisms for pathologic processes in families. Systems and Development: The Minnesota Symposia on Child Psychology, Volume 22 (Minnesota Symposia on Child Psychology Series).

[42] Spivack G, Shure MB (1976). Social Adjustment of Young Children: A Cognitive Approach to Solving Real-life Problems.

[43] Guerney B, Coufal J, Vogelsong E (1981). Relationship enhancement versus a traditional approach to therapeutic/preventative/enrichment parent-adolescent programs. J Consult Clin Psychol.

[44] Patterson GR, DeBaryshe BD, Ramsey E (1989). A developmental perspective on antisocial behavior. Am Psychol.

[45] Patterson GR (1982). Coercive Family Process.

[46] Patterson GR, Reid JB, Dishion T (1992). Antisocial Boys.

[47] Granic I, Patterson GR (2006). Toward a comprehensive model of antisocial development: a dynamic systems approach. Psychol Rev.

[48] Ary DV, Duncan TE, Duncan SC, Hops H (1999). Adolescent problem behavior: the influence of parents and peers. Behav Res Ther.

[49] Kumpfer KL, Alvarado R, Whiteside HO (2003). Family-based interventions for substance use and misuse prevention. Subst Use Misuse.

[50] Sambrano S, Springer JF, Sale E, Kasim R, Hermann J (2005). Understanding prevention effectiveness in real-world settings: the National Cross-Site Evaluation of high risk youth programs. Am J Drug Alcohol Abuse.

[51] Fothergill KE, Ensminger ME (2006). Childhood and adolescent antecedents of drug and alcohol problems: a longitudinal study. Drug Alcohol Depend.

[52] Kumpfer KL, Ashery RS, Robertson EB, Kumpfer KL (1998). Selective prevention interventions: the Strengthening Families program. Drug Abuse Prevention Through Family Interventions.

[53] Frick PJ, Christian RE, Wootton JM (1999). Age trends in the association between parenting practices and conduct problems. Behav Modif.

[54] Shelton KK, Frick PJ, Wootton J (1996). Assessment of parenting practices in families of elementary school-age children. J Clin Child Psychol.

[55] Elgar FJ, Waschbusch DA, Dadds MR, Sigvaldason N (2007). Development and validation of a short form of the Alabama Parenting Questionnaire. J Child Fam Stud.

[56] Essau CA, Sasagawa S, Frick PJ (2006). Psychometric properties of the Alabama Parenting Questionnaire. J Child Fam Stud.

[57] Moos RH, Moos BS (1984). Family Environment Scale Manual.

[58] Oliver JM, Handal PJ, Enos DM, May MJ (1988). Factor structure of the Family Environment Scale: Factors based on items and subscales. Educ Psychol Meas.

[59] Kellam SG, Rebok GW, Mayer LS, Ialongo N, Kalodner CR (1994). Depressive symptoms over first grade and their response to a developmental epidemiologically based preventive trial aimed at improving achievement. Dev Psychopathol.

[60] Kovacs M (1981). Rating scales to assess depression in school-aged children. Acta Paedopsychiatr.

[61] Saylor CF, Finch AJ, Spirito A, Bennett B (1984). The children's depression inventory: a systematic evaluation of psychometric properties. J Consult Clin Psychol.

[62] Achenbach TM, Edelbrock C (1983). Manual for the Child: Behavior Checklist and Revised Child Behavior Profile.

[63] Achenbach TM, Ruffle TM (2000). The Child Behavior Checklist and related forms for assessing behavioral/emotional problems and competencies. Pediatr Rev.

[64] Kellam SG, Ling X, Merisca R, Brown CH, Ialongo N (1998). The effect of the level of aggression in the first grade classroom on the course and malleability of aggressive behavior into middle school. Dev Psychopathol.

[65] Werthamer-Larsson L, Kellam S, Wheeler L (1991). Effect of first-grade classroom environment on shy behavior, aggressive behavior, and concentration problems. Am J Community Psychol.

[66] Kellam SG, Branch JD, Agrawal KC, Ensminger ME (1974). Mental Health and Going to School: The Woodlawn Program of Assessment, Early Intervention, and Evaluation.

[67] Dolan LJ, Kellam SG, Brown C, Werthamer-Larsson L, Rebok GW, Mayer LS, Laudolff J, Turkkan JS, Ford C, Wheeler L (1993). The short-term impact of two classroom-based preventive interventions on aggressive and shy behaviors and poor achievement. J Appl Dev Psychol.

[68] Elliott SN, Gresham FM, Freeman T, McCloskey G (1988). Teacher and observer ratings of children's social skills: validation of the social skills rating scales. J Psychoeduc Assess.

[69] Gresham F, Elliott S (1990). The Social Skills Rating System (SSRS).

[70] Dunst CJ (2017). Procedures for developing evidence-informed performance checklists for improving early childhood intervention practices. J Educ Learn.

[71] Dunst CJ, Trivette CM, Freidman S, Haywood HC (1994). Methodological consideration and strategies for studying the long term follow up of early intervention. Developmental Follow-up: Concepts, Domains, and Methods.

[72] Johnston L, O'Malley P, Bachman J (1995). Volume I secondary school students. U.S. DHHS PHS, NIH Pub. No. 95-4026.

[73] (1998). National household survey on drug abuse: Population estimates 1994. U.S. DHHS, PHS, DHHS Pub. No. (SMA) 95-3063 National Health Interview Survey: Form HIS-1A. U.S. DHHS, PHS.

[74] Cohen J (1973). Eta-squared and partial eta-squared in fixed factor ANOVA designs. Educ Psychol Meas.

[75] Ferguson CJ (2009). An effect size primer: a guide for clinicians and researchers. Prof Psychol Res Prac.

[76] Le Henanff A, Giraudeau B, Baron G, Ravaud P (2006). Quality of reporting of noninferiority and equivalence randomized trials. J Am Med Assoc.

[77] Heinrichs N, Bertram H, Kuschel A, Hahlweg K (2005). Parent recruitment and retention in a universal prevention program for child behavior and emotional problems: barriers to research and program participation. Prev Sci.

[78] Prinz RJ, Smith EP, Dumas JE, Laughlin JE, White DW, Barrón R (2001). Recruitment and retention of participants in prevention trials involving family-based interventions. Am J Prev Med.

[79] Lakens D (2013). Calculating and reporting effect sizes to facilitate cumulative science: a practical primer for t-tests and ANOVAs. Front Psychol.

[80] Lynne-Landsman SD, Bradshaw CP, Ialongo NS (2010). Testing a developmental cascade model of adolescent substance use trajectories and young adult adjustment. Dev Psychopathol.

[81] Masten AS, Desjardins CD, McCormick CM, Kuo SI, Long JD (2010). The significance of childhood competence and problems for adult success in work: a developmental cascade analysis. Dev Psychopathol.

[82] Burt KB, Obradović J, Long JD, Masten AS (2008). The interplay of social competence and psychopathology over 20 years: testing transactional and cascade models. Child Dev.

[83] Smokowski P, Corona R, Bacallao M, Fortson BL, Marshall KJ, Yaros A (2018). Addressing barriers to recruitment and retention in the implementation of parenting programs: lessons learned for effective program delivery in rural and urban areas. J Child Fam Stud.

[84] Chang R, Little TD (2018). Innovations for evaluation research: multiform protocols, visual analog scaling, and the retrospective pretest-posttest design. Eval Health Prof.

[85] Howard G, Dailey P (1979). Response-shift bias: a source of contamination of self-report measures. J Appl Psychol.

[86] Snapinn SM (2000). Noninferiority trials. Curr Control Trials Cardiovasc Med.

