# Supplementary material for: A Parenting Behavior Intervention (the Strengthening Families Program) for Families: Noninferiority Trial of Different Program Delivery Methods
Source: JMIR Pediatr Parent. 2019 Nov 18;2(2):e14751. doi: 10.2196/14751 (PMC6887825; doi:10.2196/14751)
Supplement: Multimedia Appendix 1 [file pediatrics_v2i2e14751_app1.pdf]

Table 2. Comparison of Strengthening Family Program (SFP) group norms with 3 SFP DVD conditions

Scale name and intervention settings		Sample number	Pretest, mean (SD)	Posttest, mean (SD)	Change in score	*F* score (df)	*P* value	Partial eta-square (95% CI)
**Parental involvement**						1.76 (1,3)	.15	0.01 (0.00-0.02)
	SFP[a] group norms 12 to 18	448	3.33 (0.92)	4.19 (0.70)	**0.87**	518.47 (1,447)	<.001	0.54 (0.48-0.59)
	G 1[b]—home-use DVD[c]	81	3.75 (0.66)	4.42 (0.39)	**0.67**	119.38 (1,80)	<.001	0.60 (0.46-0.69)
	G2[d]—family discussion group and DVD	18	3.46 (0.68)	4.22 (0.54)	**0.76**	31.41 (1,17)	<.001	0.65 (0.30-0.78)
	G3[e]—classroom and DVD clips	113	3.17 (0.90)	4.05 (0.69)	**0.88**	173.91 (1,112)	<.001	0.61 (0.49-0.69)
**Parental supervision**						1.86 (1,3)	.13	0.01 (0.00-0.02)
	SFP group norms 12 to 18	454	3.18 (0.72)	4.04 (0.56)	**0.86**	653.37 (1,453)	<.001	0.59 (0.54-0.63)
	G1—home-use DVD	81	3.31 (0.72)	4.35 (0.43)	**1.04**	17.48 (1,80)	<.001	0.68 (0.56-0.75)
	G2—family discussion group and DVD	27	3.21 (0.77)	3.97 (0.50)	**0.76**	37.28 (1,26)	<.001	0.59 (0.31-0.73)
	G3—classroom and DVD clips	115	3.16 (0.72)	4.09 (0.47)	**0.93**	244.03 (1,114)	<.001	0.68 (0.58-0.75)
**Parenting efficacy**						2.09 (1,3)	.10	0.01 (0.00-0.02)
	SFP group norms 12 to 18	456	3.29 (0.80)	4.14 (0.62)	**0.85**	573.61 (1,455)	<.001	0.56 (0.50-0.60)

	G1—home-use DVD	81	3.40 (0.62)	4.12 (0.46)	**0.72**	139.59 (1,80)	<.001	0.64 (0.50-0.72)
	G2—family discussion group and DVD	29	2.98 (0.60)	4.07 (0.61)	**1.09**	76.58 (1,28)	<.001	0.73 (0.52-0.82)
	G3—classroom and DVD clips	115	3.15 (0.79)	4.03 (0.60)	**0.88**	191.74 (1,114)	<.001	0.63 (0.52-0.70)
**Positive parenting**						2 (1,3)	.11	0.01 (0.00-0.02)
	SFP group norms 12 to 18	458	3.61 (0.88)	4.45 (0.58)	**0.85**	581.75 (1,457)	<.001	0.56 (0.50-0.61)
	G1—home-use DVD	81	3.74 (0.78)	4.47 (0.46)	**0.72**	138.17 (1,80)	<.001	0.63 (0.50-0.72)
	G2—family discussion group and DVD	26	3.59 (0.81)	4.44 (0.51)	**0.85**	38.62 (1,25)	<.001	0.61 (0.32-0.74)
	G3—classroom and DVD clips	117	3.40 (0.90)	4.38 (0.56)	**0.97**	193.82 (1,116)	<.001	0.63 (0.52-0.70)
**SFP parenting skills**						2.37 (1,3)	.07	0.01 (0.00-0.03)
	SFP group norms 12 to 18	448	3.31 (0.70)	3.82 (0.64)	**0.51**	401.76 (1,447)	<.001	0.47 (0.41-0.53)
	G1—home-use DVD	81	3.72 (0.55)	4.24 (0.46)	**0.53**	94.5 (1,80)	<.001	0.54 (0.39-0.64)
	G2—family discussion group and DVD	17	2.87 (0.80)	3.72 (0.70)	**0.85**	19.59 (1,16)	<.001	0.55 (0.17-0.72)
	G3—classroom and DVD clips	109	3.49 (0.69)	4.05 (0.55)	**0.56**	15.38 (1,108)	<.001	0.58 (0.46-0.67)

**Family cohesion**						5.28 (1,3)	<.001	0.02 (0.00-0.05)
	SFP group norms 12 to 18	458	3.60 (0.95)	4.37 (0.67)	**0.77**	428.12 (1,457)	<.001	0.48 (0.42-0.54)
	G1—home-use DVD	81	3.86 (0.77)	4.40 (0.47)	**0.54**	73.26 (1,80)	<.001	0.48 (0.32-0.59)
	G2—family discussion group and DVD	29	3.24 (0.75)	4.24 (0.66)	**1**	52.39 (1,28)	<.001	0.65 (0.40-0.77)
	G3—classroom and DVD clips	115	3.29 (0.99)	4.25 (0.60)	**0.96**	155.21 (1,114)	<.001	0.58 (0.46-0.66)
**Family communication**						4.3 (1,3)	.01	0.02 (0.00-0.04)
	SFP group norms 12 to 18	448	3.23 (0.76)	4.17 (0.58)	**0.94**	866.7 (1,447)	<.001	0.66 (0.61-0.70)
	G1—home-use DVD	81	2.95 (0.92)	4.19 (0.41)	**1.24**	134.06 (1,80)	<.001	0.63 (0.49-0.71)
	G2—family discussion group and DVD	27	3.32 (0.56)	4.21 (0.45)	**0.89**	66.12 (1,26)	<.001	0.72 (0.49-0.81)
	G3—classroom and DVD clips	117	3.16 (0.67)	4.10 (0.54)	**0.94**	289.99 (1,116)	<.001	0.71 (0.63-0.77)
**Family conflict**						1.59 (1,3)	.19	—
	SFP group norms 12 to 18	458	2.28 (1.01)	1.92 (0.85)	**0.36**	111.42 (1,457)	<.001	0.20 (0.14-0.26)
	G1—home-use DVD	81	2.25 (0.72)	1.82 (0.71)	**0.43**	46.04 (1,80)	<.001	0.37 (0.20-0.50)
	G2—family discussion group and DVD	26	2.89 (0.81)	2.61 (0.78)	**0.29**	13.24 (1,25)	<.001	0.35 (0.07-0.56)

	G3—classroom and DVD clips	115	2.70 (1.00)	2.19 (0.72)	**0.51**	56.36 (1,114)	<.001	0.33 (0.20-0.45)
**Family organization**						1 (1,3)	.39	0.00 (0.00-0.02)
	SFP group norms 12 to 18	454	2.84 (0.85)	3.95 (0.70)	**1.11**	815.78 (1,453)	<.001	0.64 (0.59-0.68)
	G1—home-use DVD	81	2.79 (0.73)	4.06 (0.47)	**1.26**	212.96 (1,80)	<.001	0.73 (0.62-0.79)
	G2—family discussion group and DVD	18	2.85 (0.71)	3.92 (0.54)	**1.07**	44.58 (1,17)	<.001	0.72 (0.41-0.83)
	G3—classroom and DVD clips	116	2.67 (0.82)	3.85 (0.61)	**1.19**	299.82 (1,115)	<.001	0.72 (0.64-0.78)
**Family strengths/resilience**						0.45 (1,3)	.72	0.00 (0.00-0.01)
	SFP group norms 12 to 18	446	3.29 (0.79)	4.21 (0.60)	**0.91**	815.09 (1,445)	<.001	0.65 (0.60-0.69)
	G1—home-use DVD	81	3.34 (0.74)	4.31 (0.40)	**0.98**	252.1 (1,80)	<.001	0.76 (0.66-0.81)
	G2—family discussion group and DVD	17	2.95 (0.65)	4.00 (0.59)	**1.06**	58.43 (1,16)	<.001	0.79 (0.51-0.87)
	G3—classroom and DVD clips	113	3.21 (0.78)	4.15 (0.57)	**0.94**	256.14 (1,112)	<.001	0.70 (0.60-0.76)
**Covert aggression**						8.51 (1,3)	<.001	0.04 (0.01-0.07)
	SFP group norms 12 to 18	428	1.99 (0.59)	1.75 (0.53)	**0.24**	91.88 (1,427)	<.001	0.18 (0.12-0.24)
	G1—home-use DVD	81	1.74 (0.40)	1.41 (0.27)	**0.34**	74.34 (1,80)	<.001	0.48 (0.32-0.59)

	G2—family discussion group and DVD	18	2.84 (1.06)	1.99 (0.68)	**0.85**	12.9 (1,17)	<.001	0.43 (0.08-0.64)
	G3—classroom and DVD clips	115	2.04 (0.66)	1.75 (0.45)	**0.28**	37.34 (1,114)	<.001	0.25 (0.12-0.37)
**Depression**						3.72 (1,3)	.01	0.02 (0.00-0.04)
	SFP group norms 12 to 18	442	2.19 (0.73)	1.90 (0.67)	**0.29**	116.59 (1,441)	<.001	0.21 (0.15-0.27)
	G1—home-use DVD	81	2.04 (0.47)	1.66 (0.34)	**0.38**	72.95 (1,80)	<.001	0.48 (0.32-0.59)
	G2—family discussion group and DVD	18	2.81 (0.86)	2.19 (0.82)	**0.61**	11.84 (1,17)	<.001	0.41 (0.06-0.63)
	G3—classroom and DVD clips	113	2.48 (0.77)	2.03 (0.59)	**0.44**	61.95 (1,112)	<.001	0.36 (0.22-0.47)
**Overt aggression**						7.02 (1,3)	<.001	0.03 (0.01-0.06)
	SFP group norms 12 to 18	433	2.00 (0.66)	1.67 (0.53)	**0.33**	202.75 (1,432)	<.001	0.32 (0.25-0.38)
	G1—home-use DVD	81	1.95 (0.53)	1.47 (0.34)	**0.47**	98.86 (1,80)	<.001	0.55 (0.40-0.65)
	G2—family discussion group and DVD	18	2.80 (0.96)	2.02 (0.54)	**0.78**	16.77 (1,17)	<.001	0.50 (0.13-0.69)
	G3—classroom and DVD clips	112	2.20 (0.71)	1.77 (0.50)	**0.43**	83.68 (1,111)	<.001	0.43 (0.29-0.54)
**Social behavior**						0.55 (1,3)	.65	0.00 (0.00-0.01)

	SFP group norms 12 to 18	435	3.69 (0.69)	3.99 (0.64)	**0.3**	219.67 (1,434)	<.001	0.34 (0.27-0.40)
	G1—home-use DVD	81	3.81 (0.52)	4.12 (0.42)	**0.3**	9.59 (1,80)	<.001	0.53 (0.38-0.64)
	G2—family discussion group and DVD	8	3.17 (0.81)	3.43 (0.84)	**0.26**	19.9 (1,7)	<.001	0.74 (0.18-0.86)
	G3—classroom and DVD clips	112	3.63 (0.65)	3.98 (0.57)	**0.35**	10 (1,111)	<.001	0.47 (0.34-0.57)
**Alcohol and drug use**						15.66 (1,3)	<.001	0.07 (0.03-0.10)
	SFP group norms 12 to 18	451	1.22 (0.44)	1.19 (0.44)	**0.02**	3.1 (1,450)	.08	0.01 (0.00-0.03)
	G1—home-use DVD	81	1.16 (0.30)	1.07 -0.26	**0.09**	19.61 (1,80)	<.001	0.20 (0.06-0.34)
	G2—family discussion group and DVD	18	1.59 (0.68)	1.14 (0.23)	**0.45**	11.25 (1,17)	<.001	0.40 (0.06-0.62)
	G3—classroom and DVD clips	115	1.10 (0.31)	1.06 (0.16)	**0.05**	5.03 (1,114)	.03	0.04 (0.00-0.13)
