# Supplementary material for: A Parenting Behavior Intervention (the Strengthening Families Program) for Families: Noninferiority Trial of Different Program Delivery Methods
Source: JMIR Pediatr Parent. 2019 Nov 18;2(2):e14751. doi: 10.2196/14751 (PMC6887825; doi:10.2196/14751)
Supplement: Multimedia Appendix 2 [file pediatrics_v2i2e14751_app2.pdf]

Table S.1. Comparison of Unadjusted and Adjusted Effect Sizes for SFP Non-Inferiority Trial				
**Scale Name**	**Unadjusted** η_p^2	**Adjusted 1** η_p^2	P_b	**(95% CI)**
Parental Involvement				
SFP Group Norms 12 to 16	0.54	0.48	<.001	(0.39-0.51)
G#1 - Family Viewed DVD at Home	0.60	0.54	<.001	(0.23-0.54)
G#2 – Family Discussion Group + DVD	0.65	0.62	0.02	(0.00-0.79)
G#3 – Group Class + DVD	0.61	0.56	<.001	(0.17-0.44)
Parental Supervision				
SFP Group Norms 12 to 16	0.59	0.54	<.001	(0.45-0.56)
G#1 - Family Viewed DVD at Home	0.68	0.75	<.001	(0.50-0.73)
G#2 – Family Discussion Group + DVD	0.59	0.72	<.001	(0.27-0.79)
G#3 – Group Class + DVD	0.68	0.63	<.001	(0.21-0.47)
Parenting Efficacy				
SFP Group Norms 12 to 16	0.56	0.5	<.001	(0.42-0.54)
G#1 - Family Viewed DVD at Home	0.64	0.58	<.001	(0.28-0.58)
G#2 – Family Discussion Group + DVD	0.73	0.71	<.001	(0.32-0.79)
G#3 – Group Class + DVD	0.63	0.66	<.001	(0.26-0.51)
Positive Parenting				
SFP Group Norms 12 to 16	0.56	0.51	<.001	(0.42-0.54)
G#1 - Family Viewed DVD at Home	0.63	0.64	<.001	(0.34-0.63)
G#2 – Family Discussion Group + DVD	0.61	0.74	<.001	(0.19-0.76)
G#3 – Group Class + DVD	0.63	0.63	<.001	(0.26-0.51)
SFP Parenting Skills				
SFP Group Norms 12 to 16	0.47	0.41	<.001	(0.32-0.45)
G#1 - Family Viewed DVD at Home	0.54	0.56	<.001	(0.31-0.61)
G#2 – Family Discussion Group + DVD	0.55	0.44	0.02	(0.04-0.85)
G#3 – Group Class + DVD	0.58	0.57	<.001	(0.22-0.49)
Family Cohesion				
SFP Group Norms 12 to 16	0.48	0.42	<.001	(0.34-0.47)
G#1 - Family Viewed DVD at Home	0.48	0.35	<.001	(0.07-0.37)
G#2 – Family Discussion Group + DVD	0.65	0.6	<.001	(0.19-0.73)
G#3 – Group Class + DVD	0.58	0.6	<.001	(0.31-0.56)
Family Communication				
SFP Group Norms 12 to 16	0.66	0.61	<.001	(0.53-0.63)
G#1 - Family Viewed DVD at Home	0.63	0.76	<.001	(0.51-0.74)
G#2 – Family Discussion Group + DVD	0.72	0.71	<.001	(0.40-0.84)
G#3 – Group Class + DVD	0.71	0.7	<.001	(0.33-0.57)
Family Conflict				
SFP Group Norms 12 to 16	0.20	0.18	<.001	(0.10-0.21)
G#1 - Family Viewed DVD at Home	0.37	0.39	<.001	(0.08-0.39)

G#2 – Family Discussion Group + DVD	0.35	0.31	0.16	(0.01-0.60)
G#3 – Group Class + DVD	0.33	0.23	0.002	(0.01-0.21)
Family Organization				
SFP Group Norms 12 to 16	0.64	0.6	<.001	(0.52-0.62)
G#1 - Family Viewed DVD at Home	0.73	0.77	<.001	(0.53-0.75)
G#2 – Family Discussion Group + DVD	0.72	0.54	0.001	(0.41-0.93)
G#3 – Group Class + DVD	0.72	0.72	<.001	(0.35-0.59)
Family Strengths/Resilience				
SFP Group Norms 12 to 16	0.65	0.6	<.001	(0.53-0.63)
G#1 - Family Viewed DVD at Home	0.76	0.75	<.001	(0.48-0.72)
G#2 – Family Discussion Group + DVD	0.79	0.70	<.001	(0.66-0.97)
G#3 – Group Class + DVD	0.70	0.67	<.001	(0.27-0.53)
Covert Aggression				
SFP Group Norms 12 to 16	0.18	0.15	<.001	(0.07-0.18)
G#1 - Family Viewed DVD at Home	0.48	0.56	<.001	(0.27-0.57)
G#2 – Family Discussion Group + DVD	0.43	0.32	0.006	(0.13-0.87)
G#3 – Group Class + DVD	0.25	0.16	0.002	(0.01-0.20)
Depression				
SFP Group Norms 12 to 16	0.21	0.17	<.001	(0.09-0.21)
G#1 - Family Viewed DVD at Home	0.48	0.57	<.001	(0.28-0.58)
G#2 – Family Discussion Group + DVD	0.41	0.32	0.03	(0.00-0.79)
G#3 – Group Class + DVD	0.36	0.29	<.001	(0.08-0.33)
Overt Aggression				
SFP Group Norms 12 to 16	0.32	0.28	<.001	(0.18-0.31)
G#1 - Family Viewed DVD at Home	0.55	0.64	<.001	(0.33-0.62)
G#2 – Family Discussion Group + DVD	0.50	0.41	0.02	(0.04-0.82)
G#3 – Group Class + DVD	0.43	0.36	<.001	(0.08-0.33)
Social Behavior				
SFP Group Norms 12 to 16	0.34	0.27	<.001	(0.17-0.30)
G#1 - Family Viewed DVD at Home	0.53	0.55	<.001	(0.22-0.54)
G#2 – Family Discussion Group + DVD	0.74	–[d]	–	–
G#3 – Group Class + DVD	0.47	0.43	<.001	(0.12-0.38)
Alcohol & Drug Use				
SFP Group Norms 12 to 16	0.01	0.01	0.12	(0.00-0.03)
G#1 - Family Viewed DVD at Home	0.20	0.35	<.001	(0.10-0.41)
G#2 – Family Discussion Group + DVD	0.40	0.48	0.001	(0.43-0.94)
G#3 – Group Class + DVD	0.04	0.03	0.84	(0.00-0.03)

Notes: [a]Adjusted for demographics. [b]p-value corresponds to final model with demographic measures. [d]too few cases for precise robust computation.
